# Effects of High Temperature on COVID‐19 Deaths in U.S. Counties

**DOI:** 10.1029/2022GH000705

**Published:** 2023-02-24

**Authors:** Bowen Chu, Renjie Chen, Qi Liu, Haikun Wang

**Affiliations:** ^1^ Joint International Research Laboratory of Atmospheric and Earth System Sciences School of Atmospheric Sciences Nanjing University Nanjing China; ^2^ School of Public Health Key Lab of Public Health Safety of the Ministry of Education and National Health Commission Key Lab of Health Technology Assessment Fudan University Shanghai China; ^3^ Collaborative Innovation Center of Climate Change Nanjing China; ^4^ Frontiers Science Center for Critical Earth Material Cycling Nanjing University Nanjing China

**Keywords:** COVID‐19, high temperature, mortality, The United States, SARS‐CoV‐2

## Abstract

The United States of America (USA) was afflicted by extreme heat in the summer of 2021 and some states experienced a record‐hot or top‐10 hottest summer. Meanwhile, the United States was also one of the countries impacted most by the coronavirus disease 2019 (COVID‐19) pandemic. Growing numbers of studies have revealed that meteorological factors such as temperature may influence the number of confirmed COVID‐19 cases and deaths. However, the associations between temperature and COVID‐19 severity differ in various study areas and periods, especially in periods of high temperatures. Here we choose 119 US counties with large counts of COVID‐19 deaths during the summer of 2021 to examine the relationship between COVID‐19 deaths and temperature by applying a two‐stage epidemiological analytical approach. We also calculate the years of life lost (YLL) owing to COVID‐19 and the corresponding values attributable to high temperature exposure. The daily mean temperature is approximately positively correlated with COVID‐19 deaths nationwide, with a relative risk of 1.108 (95% confidence interval: 1.046, 1.173) in the 90th percentile of the mean temperature distribution compared with the median temperature. In addition, 0.02 YLL per COVID‐19 death attributable to high temperature are estimated at the national level, and distinct spatial variability from −0.10 to 0.08 years is observed in different states. Our results provide new evidence on the relationship between high temperature and COVID‐19 deaths, which might help us to understand the underlying modulation of the COVID‐19 pandemic by meteorological variables and to develop epidemic policy response strategies.

## Key Points

- This study explores the relationship between COVID‐19 deaths and temperature by applying a two‐stage epidemiological analytical approach during the summer of 2021 in the Unites States
- The daily mean temperature is approximately positively correlated with COVID‐19 deaths nationwide, with different shapes of temperature–mortality curves noted at the regional level
- Evaluation of temperature‐mortality relationship is important in controlling COVID‐19 severity

## 1 Introduction

The COVID‐19 pandemic has triggered an unparalleled public health crisis worldwide since early 2020 (Callaway et al., 2020; Kandel et al., 2020; WHO, 2020). Up to 1 March 2022, 438,633,501 confirmed cases of COVID‐19 and 5,965,281 deaths were reported across the world, according to the statistics of the Johns Hopkins University Center for Systems Science and Engineering (Dong et al., 2020). As a global public health emergency (Mahase, 2020), COVID‐19 has deeply changed people's lives and caused a huge disruptive shock to economic development, human health and social stability (Banerjee et al., 2020; Bashir, Ma, & Shahzad, 2020; Egger et al., 2021; Meng et al., 2021; Steptoe & Di Gessa, 2021; Van Bavel et al., 2020). With the emergence and transmission of virus variants, such as Delta (CDC, 2021c; Kupferschmidt & Wadman, 2021; WHO, 2021), there remains great uncertainty worldwide regarding modifiable measures to inhibit the COVID‐19 pandemic, and identifying probable factors related to the spread and severity of COVID‐19 is crucial (Kupferschmidt & Wadman, 2021; Potvin, 2021).

Extensive studies have analyzed natural and social factors affecting the outbreak of COVID‐19 and suggested that the temperature and other meteorological factors might influence the transmission of severe acute respiratory syndrome coronavirus 2 (SARS‐CoV‐2) and human immunity (Aboura, 2022; Coccia, 2021; Kudo et al., 2019; X. Y. Liu et al., 2021; Rojas et al., 2021; Yuan et al., 2021). In winter or under cold conditions, existing research has reported that temperature is inversely correlated with COVID‐19 cases and deaths in different countries or regions (C. Guo et al., 2021; Heibati et al., 2021; Qi et al., 2020; Rosario et al., 2020; Sajadi et al., 2020). In summer or with high temperatures, however, there are more debates and contradictory findings regarding the association between temperature and COVID‐19 (Fu et al., 2021; Ismail et al., 2022; Yuan et al., 2021). On the one hand, SARS‐CoV‐2 tends to be inactive at high temperatures or under strong ultraviolet radiation in summer, resulting in a reduction of virus reproduction and infectivity, from the perspective of microbiology (Biryukov et al., 2021; Carleton et al., 2021; Ratnesar‐Shumate et al., 2020). On the other hand, heat stress can cause damage to lung tissues, combined with dehydration and circulatory disorders (Bunker et al., 2016; GonzalezAlonso et al., 1997; White, 2006), which impairs the immune system and might exacerbate the invasion of the lungs by SARS‐CoV‐2, inducing severe acute respiratory syndrome and even causing death.

The United States of America (USA) was one of the countries that suffered most severely during the COVID‐19 pandemic. By the end of September 2021, there were the largest cumulative number of confirmed cases and deaths in the USA when compared with other countries, accounting for about 19% and 14% of all confirmed cases and deaths reported worldwide, respectively (Dong et al., 2020). Worse still, extreme heat swept the contiguous states in the summer of 2021 and some states underwent the hottest or top‐10 hottest summer on record (Dolce, 2021). A considerable amount of epidemiological evidence testifies that high temperature is a risk factor for human health, associated with excess risk for mortality and morbidity (Anderson & Bell, 2011; Basu et al., 2008; Chen et al., 2018; J. Cheng et al., 2019; Gasparrini et al., 2015; Y. M. Guo et al., 2017). Nevertheless, the effects of heat on confirmed cases and deaths due to COVID‐19 are less studied.

In order to explore the influence of this heat event in summer 2021 and provide a new perspective on the associations between high temperature and COVID‐19, we conducted a systematic analysis utilizing epidemiological methods. We focused on the mortality associated with COVID‐19 and used generalized additive models linked with distributed lag non‐linear models (DLNM) to investigate the relationship between temperature and COVID‐19 deaths in the continental USA during the summer months of 2021. In order to ensure data accuracy and control fluctuations of model fitting, we selected 119 counties with large numbers of COVID‐19 deaths in the period of study. A multivariate meta‐analysis was employed to build the temperature–mortality curves at national and regional levels, and the potential social‐economic drivers (e.g., personal income and percent uninsured) behind the heterogeneity among regions were explored. We also calculated the years of life lost (YLL) due to COVID‐19 and the corresponding YLL linked with high temperature exposure.

## 2 Materials and Methods

### 2.1 Data and Study Area

The summer period was identified from June 1st to 30 September 2021 in this study. Daily COVID‐19 deaths in each county in the 4 months were collected from the public data set USAFacts.org, which is an open source of the US county‐level COVID‐19 data (USAFacts.org, 2021). USAFacts assembles the day‐to‐day county‐level cumulative number of positive cases and deaths from state and county public health websites, which is widely applied to academic research (K. J. G. Cheng et al., 2020; Zhou et al., 2021). We gathered the state‐level age distribution of COVID‐19 deaths from the website of the Centers for Disease Control and Prevention (CDC) Data Tracker, which provides weekly incident deaths per 1,00,000 population of different age groups and is updated every Saturday (CDC, 2021a). The life expectancy we refer to was presented in the US State Life Table (CDC, 2021b), which is compiled by the CDC National Center for Health Statistics to show the life expectancy of complete age period in 50 states and the District of Columbia. We adopted the US State Life Table in 2018, considering the renewal and completeness of the data.

Air quality data were collected from the US Environmental Protection Agency (EPA) (U.S.Environmental‐Protection‐Agency, 2021). The EPA provides the daily county‐level Air Quality Index (AQI) of five pollutants, namely carbon monoxide (CO), nitrogen dioxide (NO_2_), ozone, PM_2.5_ and PM_10_. The AQI is a dimensionless value serving as an indicator of severity for local air pollution and is determined based on the concentrations of the above‐mentioned five pollutants. In this study, we reverse‐calculated the daily concentration (in μg/m^3^) of pollutants from AQI information to fit our statistical analysis model.

Meteorological data sets, including daily maximum air temperature, minimum air temperature and water vapor pressure were obtained through the Oak Ridge National Laboratory (ORNL) Distributed Active Archive Center (DAAC) (Thornton et al., 2020). The data cover the area of Continental North America, Puerto Rico, and Hawaii as separate spatial layers in a Lambert Conformal Conic projection and are distributed in standardized Climate and Forecast (CF)‐compliant netCDF file formats. This data set provides daily grid information for daily weather variables with 1 km resolution and it has been updated to Daymet Version 4 on a monthly cycle. We merged the grid values into each county by judging whether the grid points were within the borderline of the selected county and then took an average. The daily mean temperature was calculated as the average of the daily maximum and minimum temperatures. Daily relative humidity (RH) was obtained from the following approximate equations using the Clausius–Clapeyron relation (Shaman & Kohn, 2009; Wallace & Hobbs, 2006):

(1) $$\ln\frac{e_{s}\left(T\right)}{e_{s}\left(T_{0}\right)}\approx \frac{L_{v}M_{w}}{1000R^{*}}\left(\frac{1}{T_{0}}-\frac{1}{T}\right)$$

(2) $$RH\approx e/e_{s}\times 100\%$$

where *L*_*v*_ is the latent heat of water evaporation per unit mass; *M*_*w*_ is the molar mass of water; *e*_s_(*T*) is the saturated vapor pressure of water at temperature *T*, and here we took the daily mean temperature; *T*_0_ is the temperature of an ice–water mixture under standard atmospheric pressure, namely 273.15 K.

County‐specific socio‐economic and demographic data were mainly retrieved from the CDC Data Tracker and the personal income per capita was from the Bureau of Economic Analysis (BEA) of the United States Department of Commerce (CDC, 2021a; U.S.Bureau‐of‐Economic‐Analysis, 2021). We chose the Oxford COVID‐19 Government Response Tracker (OxCGRT) Government Response Index as an indicator that reflected the state governmental policy responses to the spread of COVID‐19 (Hale et al., 2021). The OxCGRT aggregates 23 kinds of policy‐response strategies such as closures or restrictions (e.g., school and workplace closures, cancellation of public events, restrictions on gatherings), economic policies (e.g., income support, international aid), health system policies (e.g., COVID‐19 testing policy, emergency investments into healthcare) and, most recently, vaccination policies (e.g., the cost of vaccination to the individual). Those different policy responses are assembled into the OxCGRT Government Response Index (from 0 to 100) to reflect how many related policies a government has implemented, and to what degree (Hale et al., 2022). Generally, the more stringent policy responses a country or regional government enacts, the higher the OxCGRT Government Response Index will be.

After collecting the above‐mentioned data sets, we first selected counties in which there was, on average, more than one death per day in the study period, to reduce statistical inaccuracy. Following this, we checked the meteorological and air quality information and excluded counties without comprehensive data. The locations of counties with greater than one death per day on average are shown in Figure S1 of the Supporting Information S1, and the counties finally chosen for the study with data availability are presented in Table S1 of the Supporting Information S1.

### 2.2 Statistical Analysis

When building the relationship between daily temperature and COVID‐19 deaths, we used a two‐stage approach. In the first stage, we used a quasi‐Poisson generalized linear model (GLM) linked with a distributed lag non‐linear model (DLNM) to estimate county‐specific temperature–death associations (Gasparrini et al., 2010). Regarding the setting of the dependent variable, the daily deaths associated with COVID‐19 has been a regular choice in previous studies. However, we found that, for many counties, there were some days with zero or even a negative number of COVID‐19 deaths due to delays or faults in data acquisition and aggregation (Zhou et al., 2021), which would not satisfy the Poisson distribution in numerical simulations. To address this challenge, we calculated the 7‐day cumulative COVID‐19 deaths in one county and set it as the dependent variable in our model. This represents a trade‐off to eliminate negative counts of death and abate fitting deviation.

In order to describe the nonlinear and delayed relationships between temperature and mortality flexibly, a cross‐basis function in DLNM was introduced in the basic model. The cross‐basis function was defined using a quadratic B‐spline for the exposure response, with two internal knots set at the 50th and 90th percentiles of county‐level temperature distribution, because the exposure–response characteristics might be different among counties at various geographic locations. We then used a natural cubic spline function with 2 degrees of freedom (df) for the lagged response with a maximum of a one‐day lag to show immediate effects of heat. Referring to previous evidence about environmental factors and COVID‐19 (Y. L. Ma et al., 2020; Rojas et al., 2021; Sera et al., 2021; Xu et al., 2022), some potential confounding covariates were also adjusted in the GLM regression model. We employed linear terms for PM_2.5_ and diurnal temperature variation (TV), and a natural cubic spline of daily RH with 3 df to control confounding effects. A natural cubic spline function of time with 2 df was considered to adjust the long‐term trend. We took the OxCGRT Government Response Index with a 10‐day delay into the model to indicate the adjustment of the state policy response to the COVID‐19 pandemic. Specifically, we did not add dummy variables to represent day of the week (DOW) because 7‐day cumulative deaths associated with COVID‐19 was taken as the dependent variable and it would filter the DOW effect. The formula of our model is as follows:

(3) $$\begin{array}{lll} \log\left(7days\_{death}_{i,t}\right) & = & \alpha_{i}+cb\left({Tmean}_{i,t}\right)+\beta_{1,i}\cdot {TV}_{i,t}+\beta_{2,i}\cdot {PM}_{2.5,i,t}+ns\left({RH}_{i,t},{df}_{1}\right)+ns\left({time}_{i,t},{df}_{2}\right)\\ & & +{OxfordGRI}_{i,t-10} \end{array}$$

Where *i* and *t* indicates the county and day of observation; 7day_death_*i*,*t*_ denotes 7‐day cumulative COVID‐19 deaths from day *t* to day *t + 6*; *α*_*i*_ denotes the baseline intercept; cb(*T*mean_*i*,*t*_) indicates the cross‐basis function of daily mean temperature distribution. We placed 50th percentiles of temperature as the center of the cross‐basis function. The relative risk (RR) and corresponding 95% confidence interval were reported based on this setting.

In the second stage, we employed a multivariate meta‐analysis to pool the county‐level evaluations in order to analyze the national and regional nonlinear temperature–death associations (Gasparrini et al., 2012). We took county‐specific parameters attained from the first analysis stage as outcomes for the multivariate meta‐analysis. The fitting of the meta‐analyses involved a random effects model by maximum likelihood. Considering the diversity of temperature ranges for counties due to geographic disparity, we introduced relative temperature terms as an alternative when estimating the pooled temperature–mortality relation. The relative temperature terms were the percentiles of temperature distribution rather than the absolute values. This setting contributed to reduction of heterogeneity and assessment of pooled exposure–response effects. The heterogeneity across counties was tested and reported by the Cochran *Q* test and *I* ^2^ statistic (Higgins & Thompson, 2002).

We unfolded a univariable regression model to derive the correlations between county‐specific characteristics and mortality associated with COVID‐19. These county‐level factors were the 4‐month mean temperature, 4‐month mean diurnal TV, the 4‐month mean RH, personal income, percentage of vaccination, population density, percentage of people uninsured, percentage of people over 65 years and the OxCGRT Government Response Index.

With regard to the YLL caused by COVID‐19, the state‐specific age distributions of COVID‐19 deaths were downloaded from the CDC Data Tracker website (CDC, 2021a). These data indicate weekly COVID‐19 mortality in 10 different age groups: 0–4, 5–11, 12–15, 16–17, 18–29, 30–39, 40–49, 50–64, 65–74, and 75+ years. The US State Life Table from the CDC National Center for Health Statistics was applied to determine the remaining expected life by age (CDC, 2021b). As the age intervals are divided into every year (i.e., 0–1, 1–2, 2–3…98–99, 99–100, and 100+) in the US State Life Table, we calculated the YLL for COVID‐19 death through the following equations:

(4) $$L_{i}=\frac{\sum_{a=lower}^{a=upper}n_{a}\cdot l_{a}}{\sum_{a=lower}^{a=upper}n_{a}}$$

(5) $${YLL\_per\_death}_{s}=\frac{\sum_{i=1}^{i=10}L_{i}\cdot N_{i}}{\sum_{i=1}^{i=10}N_{i}}$$

Where *L*_*i*_ denotes the expected life of each age group (*i* is from 1 to 10) in the CDC Data Tracker; *l*_*a*_ denotes the life expectancy at each age provided in US State Life Table; *n*_*a*_ denotes the number of people living at each age; upper and lower denote the maximum and minimum age of each age group; *Ni* denotes the number of deaths in each age group attributed to COVID‐19; YLL_per_death_*s*_ denotes the average YLL of a COVID‐19 death in the state *s*.

Finally, we calculated the YLL per COVID‐19 death attributable to high temperatures. In the first stage of building the temperature–death relationship, we set the 50th temperature percentile as the benchmark and chose a RR in the 90th percentile as the heat threshold to explore the corresponding YLL caused by high temperatures. The overall heat effects were estimated by the sum of the contributions of each temperature exceeding the heat threshold in our study period. The equation is written as follows (T. Liu et al., 2021; Majdan et al., 2017):

(6) $${AYLL}_{i}=\frac{{YLL\_per\_death}_{s}}{D_{total}}\cdot \sum_{T=t_{percentile=90th}}^{T=t_{percentile=100th}}{AD}_{i,T}\cdot {Freq}_{T}$$

Where AYLL_*i*_ is the corresponding attributable YLL of COVID‐19 death due to high temperatures in county *i*; AD_*i*,*T*_ is the YLL attributable to each temperature percentile *T* in county *i*; Freq_*T*_ denotes the frequency of each temperature percentile *T*; *D*_total_ is the total count of COVID‐19 deaths over the study period; YLL_per death_*s*_ denotes the average YLL of a COVID‐19 death in state *s* in which county *i* is located, and this value can be obtained from Equations 4 and 5.

### 2.3 Sensitivity Analysis

To check the robustness of our findings in the main model, sensitivity analyses were conducted by: (a) alternating internal knots set at the 50th and 75th percentiles of temperature; (b) changing the df of RH from 3 to 2 and the df of time from 2 to 1 and 3; (c) replacing daily mean temperature with daily maximum temperature; (d) taking 7‐day weighted cumulative COVID‐19 deaths and setting more weight in the first 3 days in view of a decreasing effect of heat over time (Ye et al., 2012); (e) adjusting maximum temperature lag from 1 to 3; (f) changing the RH to absolute humidity; (g) replacing the exposure‐response basis from B‐spline to natural spline and fourth‐order polynomials; (h) adding the variations of vaccination rates and (i) adding daily maximum 8‐hr ozone for an additional setting. Compared with the main model, the last two sensitivity analyses were conducted only on a partial sample of counties because the data of ozone and vaccination rates were not available in all of the research areas.

We used MATLAB2021b to preprocess the data and R software (version 4.1.1, R Project) to perform all the analyses, with the “dlnm” package to create the DLNM model and “mvmeta” package to conduct the meta‐analysis (Gasparrini, 2011; Gasparrini & Armstrong, 2013).

## 3 Result

### 3.1 Descriptive Statistics

As shown in Table 1, the average daily mean temperature, maximum temperature, minimum temperature, RH, PM_2.5_ concentrations and diurnal temperature variability was 25.1°C, 31.0°C, 19.2°C, 0.603, 12.3 μg/m^3^, and 11.8°C, respectively. Among 119 counties with large COVID‐19 death counts in this study, 28 counties lacked daily concentrations of PM_2.5_, therefore the statistics for these 28 counties were not included. For the death data, the mean 7‐day cumulative death count associated with COVID‐19 was 18.6 (from 0 to 287) for each county, and there were on average 313 COVID‐19 deaths in one county during the study period, with the largest count (2,175) in Harris County, TX and the lowest (122) in Guilford County, NC (Table S1 in Supporting Information S1). Social and economic characteristics of these counties are presented in Table S2 of the Supporting Information S1.

**Table 1:** Descriptive Data on COVID‐19 Deaths, Meteorological Factors and PM2.5 in the Studied Counties

	Mean (SD)	Maximum	75th percentile	Median	25th percentile	Minimum
7‐Day cumulative deaths	18.6 (29.4)	287.0	22.0	9.0	4.0	0.0
Daily mean temperature	25.1 (4.0)	37.8	27.9	25.6	22.8	7.3
Daily maximum temperature	31.0(4.3)	45.5	33.7	31.3	28.5	10.0
Daily minimum temperature	19.2(4.5)	30.3	22.6	20.1	16.3	1.3
Diurnal temperature variability	11.8 (3.7)	24.7	14.2	11.5	9.3	2.0
Daily RH	0.603 (0.193)	0.918	0.732	0.679	0.546	0.169
Daily PM_2.5_	12.3 (14.4)	509.0	14.3	9.6	6.7	0.0

### 3.2 The Associations Between Temperature and COVID‐19 Deaths

**Figure 1:**
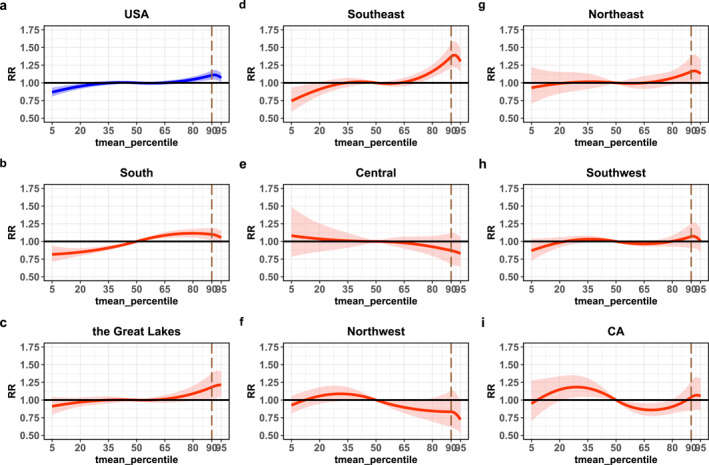
Overall cumulative temperature–mortality associations from multivariate meta‐analysis by country and region. The state groups are shown on the titles, and the vertical dashed lines represent the 90th percentiles of the temperature distribution.

Figure 1 illustrates the pooled average temperature–mortality relationship across eight regions and nationwide from the multivariate meta‐analysis. The results showed different shapes of curves, represented by diverse fluctuations and even reverse high temperature effects. The pooled curve for all counties approximately presented a slowly increasing trend (Figure 1a), indicating that high temperatures are nonlinearly associated with excess COVID‐19 death risk. The regional curves also showed roughly rising links, except for southwestern and central regions. However, the national and some regional curves displayed a downtrend when the percentile of daily mean temperature exceeded approximately the 90th. In order to exclude the influence of knot setting, we alternated internal knots from 90th to 75th percentiles of temperature in sensitivity analysis and also found the downtrend (Figure S2 in Supporting Information S1).

Table 2 summarizes the effect of high temperature (90th percentile of mean temperature distribution) relative to median of mean temperature on mortality nationwide and in eight regions. The heat effect was statistically significant at national level, with RR of 1.108 (95% CI: 1.046, 1.173). There were also statistically significant relationships in the south, southeast, northeast, and Great Lakes regions, with the greatest estimated RR of 1.340 (95% CI: 1.209, 1.552) in the southeast region. In contrast, effects of heat showed a negative change of excess risk in the northwestern and central regions, although the negative effects did not meet statistical significance, with RR of 0.834 (95% CI: 0.614, 1.133) and 0.871 (95% CI: 0.678, 1.120), respectively.

**Table 2:** Regional Division in Multivariate Meta‐Analysis and Corresponding RR (95% Confidence Interval)

State group	County number	RR: *T*mean 90th versus 50th
USA	91	1.108 (1.046, 1.173)
South	23	1.100 (1.016, 1.190)
The Great Lakes	8	1.182 (1.013, 1.381)
Southeast	21	1.370 (1.209, 1.552)
Central	8	0.871 (0.678, 1.120)
Northwest	7	0.834 (0.614, 1.133)
Northeast	7	1.159 (0.975, 1.378)
Southwest	8	1.069 (0.910, 1.257)
CA	9	1.040 (0.854, 1.266)

*Note*. Here RR was defined as the relative risk in the 90th percentile of mean temperature compared with the median temperature to reflect high temperature effects.

### 3.3 Univariable Regression Results and Heterogeneity Analysis

**Figure 2:**
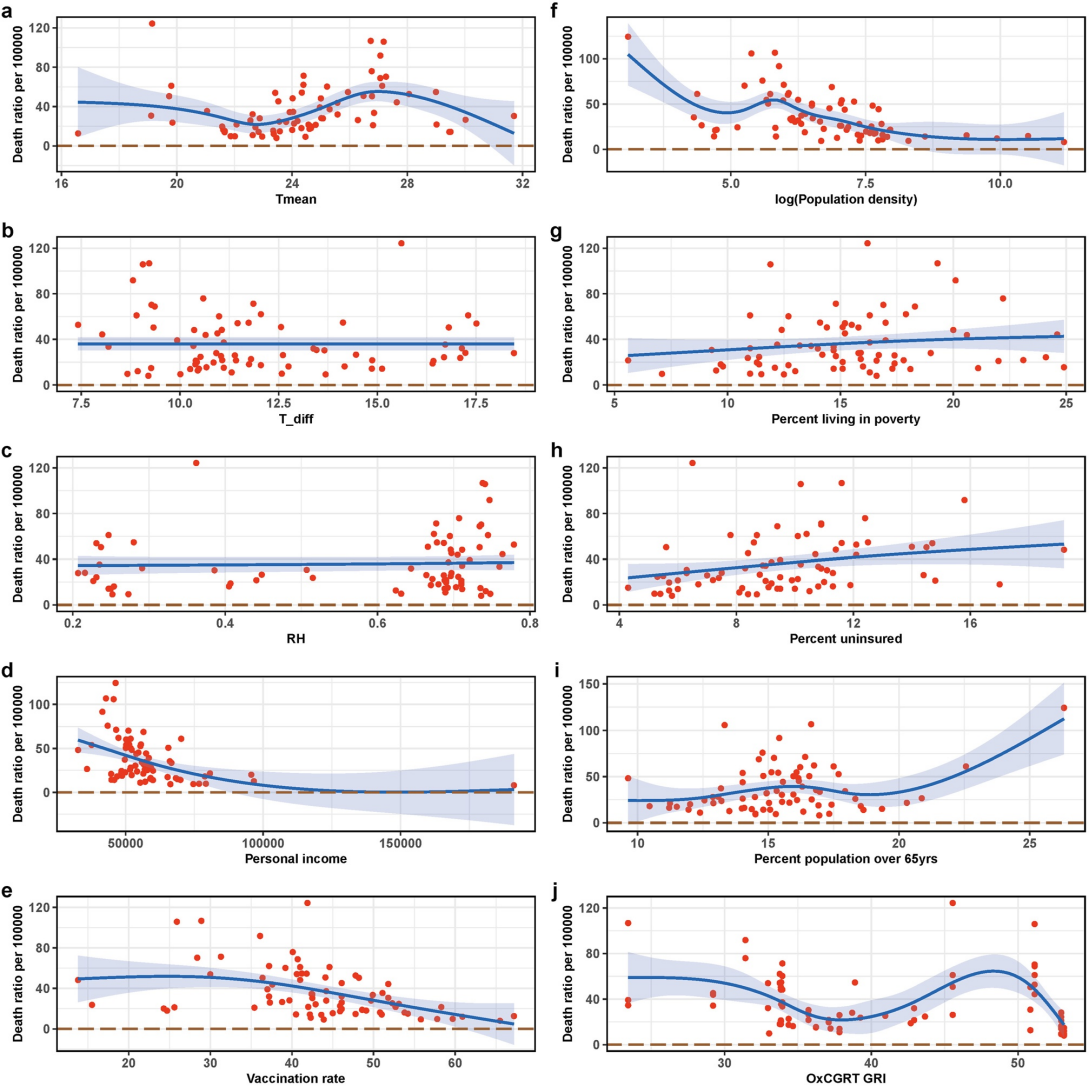
Results of univariable regression between the mortality associated with COVID‐19 and other county‐specific characteristics. The formula used in the regression curves was the cubic spline of *X*‐axis variables. Note that the *X*‐axis variable is the logarithmic value of population density in panel (f).

Figure 2 demonstrates the results of univariable regression analysis of the mortality associated with COVID‐19 linked with other county‐specific characteristics. We noticed an increased COVID‐19 mortality in counties with a higher percentage of uninsured, percentage of poverty, and percent population over 65 years of age. A decreased association was observed with personal income, vaccination rate and population density. Mean temperature and OxCGRT Government Response Index were not found to have clear relationships with mortality. Among these social‐economic characteristics, personal income, vaccination rate and population density were highly correlated with each other (*p*‐value < 0.001), and the percentages of poverty and population over 65 years old have strong relationships with the percentage of uninsured (Tables S3 and S4 in Supporting Information S1).

Results from the analysis of heterogeneity are presented in Table 3. In the meta‐analytical model without meta‐predictor, the *I* ^2^ statistics in the overall temperature–mortality association between counties was 60.6% (Cochran *Q* test *p*‐value < 0.001), and a large amount of heterogeneity was explained by state differences, as indicated by the decline in the *I* ^2^ statistics when indicators of state were added to the meta‐analysis. The county‐specific average of summer temperature and uninsured percentage also explained a limited part of the residual heterogeneity. In the Cochran *Q* test, evidence of heterogeneity was found for each model (*p*‐value < 0.001).

**Table 3:** Results of Heterogeneity Analysis in National Meta‐Regression Models

	*I* ^2^ and *Q* test	Predictors					
		Intercept only	*T*mean	Personal income	Percent uninsured	State	Full predictors
USA	*I* ^2^	60.6%	60.4%	60.8%	58.8%	51.3%	51.9%
	*Q* test	<0.001	<0.001	<0.001	<0.001	<0.001	<0.001
	Wald test	–	0.111	0.595	<0.001	<0.001	

*Note*. Referring to the univariable regression results and Spearman's correlation (Figure 2, Tables S3 and S4 in Supporting Information S1), mean temperature, personal income, percentage uninsured and state were selected as meta‐predictors. The Wald test on the significance of each meta‐predictor explains variations in national cumulative temperature–mortality curves. The Cochran *Q* test explains heterogeneity and *I* ^2^ statistics explain residual heterogeneity.

### 3.4 Effects of Heat on YLL Per COVID‐19 Death

**Figure 3:**
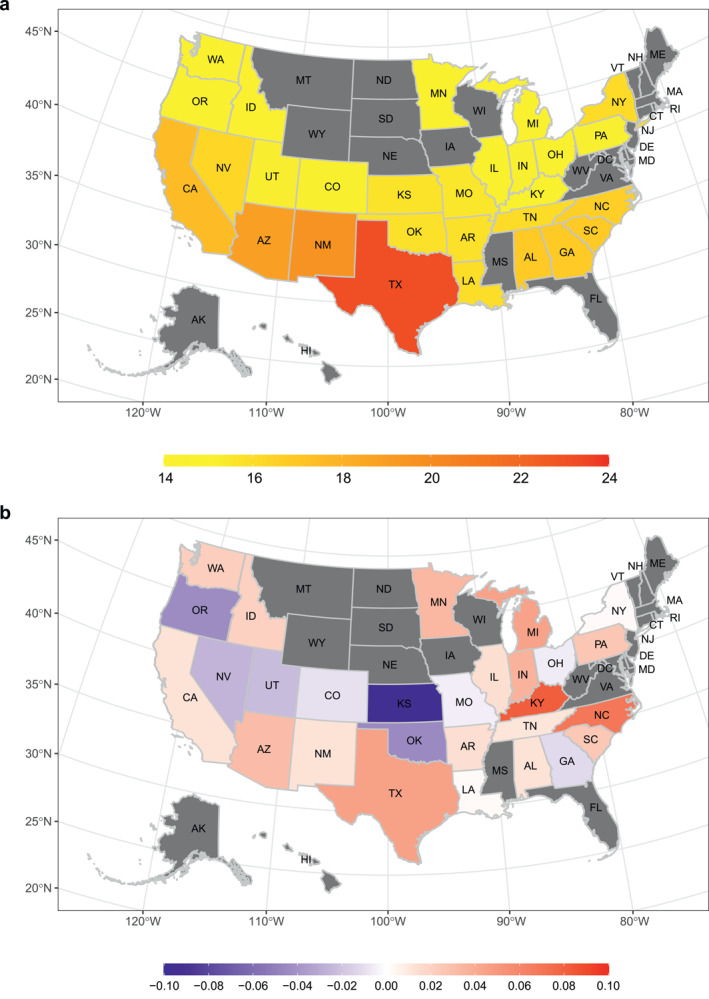
Spatial distribution of years of life lost (YLL) per COVID‐19 death (a) and YLL per COVID‐19 death attributable to high temperatures (b) at state level in the USA. In panel (b), positive and negative values represent promoting and alleviating attribution of high temperature to YLL per COVID‐19 death, respectively.

We noticed that the states with higher YLL per death were generally located in the south of the USA (Figure 3a). Texas had the largest YLL per death (23.13), and Michigan had the lowest value (14.36). Distinct differences among states were also found in the magnitude and direction of heat effects (Figure 3b). Increasing YLL effects of high temperature were noticed in most states, especially those located in the east and south of the USA. The highest YLL value per COVID‐19 death attributable to heat was found in Kentucky (0.08), and the lowest value was in Kansas (−0.10).

### 3.5 Sensitivity Analysis

Temperature–mortality relationships and the RR for heat (90th vs. 50th percentiles of temperature) in the sensitivity analyses are shown in Figures S2–S13 and Table S5 in Supporting Information S1. We found similar nonlinearly increasing trends in the national exposure–response curves when compared with the main model. The values of RR were all significantly larger than 1 except for the model in which the df of time was changed from 2 to 3.

## 4 Discussion

In this study, we enrolled 119 counties in the USA with a large number of COVID‐19 deaths during the summer months of 2021 to estimate the association between high temperature and COVID‐19 mortality. Generalized linear model combined with DLNM and multivariate meta‐analysis were adopted to depict pooled temperature–death curves at the national or regional level. We introduced univariable regression and heterogeneity tests to explore the source of heterogeneity in the temperature–mortality relationship. Compared with previous literature about meteorological factors and COVID‐19 (Aboura, 2022; C. Guo et al., 2021; Heibati et al., 2021; Qi et al., 2020; Rosario et al., 2020; Yuan et al., 2021), we focused on high temperature and further explored the YLL per COVID‐19 death attributable to high temperatures, which helped us to better understand the effect of heat exposure in reducing life expectancy.

We found that there were nonlinear correlations between temperature and risk of COVID‐19 death nationwide, which generally showed a positive trend, indicating that high temperatures may contribute to the mortality associated with COVID‐19. An approximately 10.8% increase in cumulative COVID‐19 deaths in the next 7‐day period was significantly correlated with the growth in daily mean temperature from the 50th percentile to the 90th percentile of the temperature distribution. Referring to the outcomes of sensitivity analysis, this positive relationship was relatively robust. At a regional level, most of the simulations displayed positive associations between increases in temperature and excess COVID‐19 deaths. However, we also found large spatial heterogeneity across regions, and even evidence of negative effects of heat on COVID‐19 deaths in some regions. With regard to YLL per COVID‐19 death attributable to heat, we also noticed large spatial diversity within each state, and the value merged at national level was 0.02 years, which is in a reasonable range when compared with other diseases (T. Liu et al., 2021).

Several previous studies on the relationships between temperature and the COVID‐19 pandemic have reported a positive correlation in different regions when the temperature reached a relatively high value, which is consistent with our results. For instance, Yuan et al. (2021) collected data for the whole of 2020 across 188 countries and found that the daily mean temperature showed a weak positive correlation with the number of daily cases of COVID‐19 when the temperature was above 21°C. Ismail et al. (2022) incorporated a data set from six major cities in the Kingdom of Saudi Arabia and observed that daily numbers of confirmed cases of COVID‐19 had a positive relationship with temperature between 23 and 34.5°C, and a higher number of deaths was associated with increasing temperature above 28.7°C. Bashir, Ma, Bilal et al. (2020) analyzed 2 months of data from spring 2020 in New York City and concluded that there was a significant positive correlation of daily average temperature with the total number of COVID‐19 cases and mortality. Sera et al. (2021) carried out a cross‐sectional analysis incorporating information from 409 cities in 26 countries and depicted a slow increase in the effective reproduction number of SARS‐CoV‐2 when the mean temperature was above 20°C.

From biological and physiological perspectives, some evidence supports the findings of our studies. The human body will accelerate blood flow and sweat secretion to control internal temperature with the increase of environmental temperature (Kenny & Jay, 2013), which can aggravate cardiac burden and induce dehydration (Boyette & Manna, 2019; GonzalezAlonso et al., 1997; Rowell, 1983). Cardiovascular pressure and deficits of water may lead to dysfunction of the respiratory and immune systems, combined with the direct damage caused by heat stress to cells and tissues (White, 2006; Zanobetti et al., 2012). Vulnerable lung tissues and weak immunity can reduce immune responses to SARS‐CoV‐2 infection and exacerbate viral harm to the lungs. Eventually, patients will develop severe acute respiratory syndrome and possibly die if timely and effective treatment is not provided. New immunological evidence has indicated that high environmental temperature could dampen virus‐specific CD8+ T cells and antibody production to inhibit the response of immune system (Moriyama & Ichinohe, 2019). The change of human behavior in a hot environment could also increase the risk of COVID‐19 spread (Fares, 2013). For example, people will spend more time in enclosed rooms to escape outdoor heat (Sera et al., 2021), and tend to take off their masks because masks may restrict the evaporation of sweat and feel unpleasant on the face in hot conditions. Interestingly, our results showed that the national curve had a weakly decreasing trend when the daily mean temperature approximately exceeded the 90th percentile, although the RR was still significantly greater than 1. This result may be explained by the fact that SARS‐CoV‐2 is more prone to inactivation and loss of infectivity in extreme heat and under intense ultraviolet exposure (Biryukov et al., 2021; Carleton et al., 2021; Dabisch et al., 2021; Ratnesar‐Shumate et al., 2020).

Some published investigations demonstrated negative relationships between temperature and COVID‐19 confirmed cases or deaths during the early period of the COVID‐19 outbreak (C. Guo et al., 2021; Qi et al., 2020; Rosario et al., 2020; Wu et al., 2020). In addition to the geographical heterogeneity and variations in statistical methodology, a possible explanation might be the different selection of research time. The research time window in early studies was normally set from the outbreak of COVID‐19 locally, which occurred in the winter or early spring of 2020 in the northern hemisphere. Microbiological evidence showed that some kinds of infectious virus, such as influenza, survive longer and remain more stable in a cold and dry environment (Lowen & Steel, 2014). In addition, the immunity of the host may decline so that they become more susceptible to virus infection in cold weather (Kudo et al., 2019; Lowen et al., 2007). Recent studies carried out on a long temporal scale or over a complete seasonal cycle have found nonlinear associations between temperature and the COVID‐19 pandemic (Fu et al., 2021; Ismail et al., 2022; Y. Q. Ma et al., 2021; Yuan et al., 2021). In addition, the emergence and transmission of viral variants such as the Delta variant adds difficulty and uncertainty in the analysis of time series (Kupferschmidt & Wadman, 2021). Therefore, there remains uncertainty, and there is scientific value in exploring the spread and lethality of COVID‐19 under different temperature conditions, especially high temperature conditions, in regions with diverse climatic environments and levels of socioeconomic development.

There are some strengths in the design of the statistical models in our study: (a) The selection of 7‐day cumulative counts of COVID‐19 death as a dependent variable eliminates days with zero or negative counts due to unavoidable delays or faults in data collection, as well as smoothing weekly effects. (b) The choice of percentiles of local temperature distribution was aimed at controlling geographical differences in temperature. (c) Quasi‐Poisson GLM linked with a DLNM was used to build nonlinear associations between temperature and mortality and better reflect correlations. (d) Other meteorological conditions, air quality and policy interventions were treated as confounding factors to adjust for their potential influence on the results.

However, several limitations of this study should be acknowledged. First, similar to previous studies, it was an ecological study at county‐specific level, so the results cannot avoid the individual‐level bias and might introduce ecological fallacy (Gasparrini & Armstrong, 2010; Peng et al., 2022). Second, existing studies have also found an influence of ultraviolet exposure on the COVID‐19 pandemic (Carleton et al., 2021), but we did not include ultraviolet radiation due to data unavailability in the study area. Third, the selection of counties with large counts of COVID‐19 deaths could still, more or less, introduce bias in the results of the national meta‐regression. For example, more counties were in the south of the USA, and counties with large COVID‐19 deaths might have higher infectious dynamism. Finally, we noticed the occurrence of more virus variants (e.g., Omicron), and considering prior deaths of susceptible people, vaccine effectiveness and other factors, the relationships between temperature and COVID‐19 in future hot seasons might show different characteristics, which deserves analyses in prospective studies.

## 5 Conclusion

In summary, utilizing a systematic epidemiological approach, we found an approximately positive relationship between daily mean temperature and mortality associated with COVID‐19 in US counties with a large number of deaths in the summer of 2021. When daily mean temperature rose to the 90th percentile from the local median, an increase of 11% in the 7‐day cumulative number of COVID‐19 deaths was observed. We also found an effect of high temperature with an increasing burden of 0.02 YLL per COVID‐19 death nationwide. Our results suggest that temperature plays a role in the mortality associated with COVID‐19, with spatial differences, and that effects of heat might increase the risk of COVID‐19 mortality. This study could provide some scientific information allowing policymakers to design more regional specific measures to better control the COVID‐19 pandemic in different seasons.

## Conflict of Interest

The authors declare no conflicts of interest relevant to this study.

## Supporting information

Supporting Information S1

## Data Availability

The data that support the findings of this study are all available from publicly available sources. Daily COVID‐19 deaths in each county are collected from the public data set USAFacts.org and available at https://usafacts.org/visualizations/coronavirus-covid-19-spread-map/. The state‐level age distributions of COVID‐19 deaths are from the website of the CDC Data Tracker and can be access at https://covid.cdc.gov/covid-data-tracker/#cases-deaths-testing-trends. The life expectancy we refer to is presented in the US State Life Table at https://www.cdc.gov/nchs/fastats/life-expectancy.htm. Air quality data are collected from the US Environmental Protection Agency (EPA) at the website https://www.epa.gov/outdoor-air-quality-data/air-quality-index-daily-values-report. Meteorological data sets are obtained through the Oak Ridge National Laboratory (ORNL) Distributed Active Archive Center (DAAC) via https://daac.ornl.gov/DAYMET/guides/Daymet_Daily_V4.html. County‐specific socio‐economic and demographic data are mainly retrieved from the CDC Data Tracker and the personal income per capita was from the Bureau of Economic Analysis (BEA) of the United States Department of Commerce and available at https://covid.cdc.gov/covid-data-tracker/#demographicsovertime and https://apps.bea.gov/itable/index.cfm, respectively. The OxCGRT Government Response Index is access in *Data availability* part of the article via https://doi.org/10.1038/s41562-021-01079-8.
