# Supplementary material for: Effects of High Temperature on COVID‐19 Deaths in U.S. Counties
Source: Geohealth. 2023 Feb 24;7(3):e2022GH000705. doi: 10.1029/2022GH000705 (PMC9958002; doi:10.1029/2022GH000705)
Supplement: Supplementary file 1 — Supporting Information S1 [file GH2-7-e2022GH000705-s001.pdf]

Supplemental Material

Effects of high temperature on COVID-19 deaths in U.S. counties

Bowen Chu [1], Renjie Chen [2], Qi Liu [1, 3], Haikun Wang [1, 3, 4]*

[a] Joint International Research Laboratory of Atmospheric and Earth System Sciences, School of Atmospheric Sciences, Nanjing University, Nanjing 210023, China

[b] School of Public Health, Key Lab of Public Health Safety of the Ministry of Education and National Health Commission Key Lab of Health Technology Assessment, Fudan University, Shanghai, China

[c] Collaborative Innovation Center of Climate Change, Jiangsu Province, Nanjing 210023, China

[d] Frontiers Science Center for Critical Earth Material Cycling, Nanjing University, Nanjing, 210023, China

* Email address: wanghk@nju.edu.cn;

Table of Contents

Table S1. List of counties included in the statistical analysis and total number of COVID-19 deaths in study period.

Table S2. Socio-economic characteristics of counties in the study. Note that personal income, population density, percent uninsured, percent of poverty and percent population over 65 years of each county were the statistical data in 2020. Data of cumulative vaccination rate was taken at August 1st, 2021 (median date in the study) and OxCGRT Government Response Index of counties was calculated as the average in study time.

Table S3. Spearman's rank order correlation coefficients between socio-economic characteristics of counties in study.

Table S4. Significance tests for spearman's rank order correlation between socio-economic characteristics of counties in study.

Table S5. The results of RR for heat (90th vs 50th percentiles of temperature distribution) with 95%CI in main model and sensitivity analysis.

Figure S1. Geographical distribution of the counties with large number of COVID-19 deaths in the U.S. and spatial division in the multivariate meta-analysis. The yellow triangle represents the location of the counties with large counts of COVID-19 deaths in the study period, and the grey areas represent that no county is located in these states.

Figure S2. Overall cumulative temperature–mortality associations from multivariate meta-analysis by country and regions in sensitivity analysis (setting internal knots at the 50th and 75th percentiles of temperature compared with main model).

Figure S3. Overall cumulative temperature–mortality associations from multivariate meta-analysis by country and regions in sensitivity analysis (adjusting df of RH from 3 to 2 compared with main model).

Figure S4. Overall cumulative temperature–mortality associations from multivariate meta-analysis by country and regions in sensitivity analysis (adjusting df of time from 2 to 1 compared with main model).

Figure S5. Overall cumulative temperature–mortality associations from multivariate meta-analysis by country and regions in sensitivity analysis (adjusting df of time from 2 to 3 compared with main model).

Figure S6. Overall cumulative temperature–mortality associations from multivariate meta-analysis by country and regions in sensitivity analysis (replacing daily mean temperature with daily maximum temperature compared with main model).

Figure S7. Overall cumulative temperature–mortality associations from multivariate meta-analysis by country and regions in sensitivity analysis (taking 7-day weighted cumulative deaths of COVID-19 as dependent variable compared with main model). The weighting factors were taken 1.4 in the deaths of first three days and 0.7 in the deaths of last four days to keep the sum of factors equal to 7.

Figure S8. Overall cumulative temperature–mortality associations from multivariate meta-analysis by country and regions in sensitivity analysis (adjusting maximum temperature lag from 1 to 3 compared with main model).

Figure S9. Overall cumulative temperature–mortality associations from multivariate meta-analysis by country and regions in sensitivity analysis (changing the RH to AH).

Figure S10. Overall cumulative temperature–mortality associations from multivariate meta-analysis by country and regions in sensitivity analysis (replacing the exposure-response basis from B-spline to natural spline).

Figure S11. Overall cumulative temperature–mortality associations from multivariate meta-analysis by country and regions in sensitivity analysis (replacing the exposure-response basis from B-spline to fourth-order polynomials).

Figure S12. Overall cumulative temperature–mortality associations from multivariate meta-analysis by county A) without daily changes of vaccination rates and B) with daily changes of vaccination rates in sensitivity analysis. Note that the sensitivity analyses were conducted only on partial counties compared to the main model since updating data of vaccination rates was not available in Texas.

Figure S13. Overall cumulative temperature–mortality associations from multivariate meta-analysis by country A) with daily ozone and B) without daily ozone in sensitivity analysis. Note that the sensitivity analyses were conducted only on partial counties compared to the main model since ozone data was not available in all of the research areas (81 counties with data).

Supplementary Tables

Table S1. List of counties included in the statistical analysis and total number of COVID-19 deaths in study period.

County FIPS	County	State	COVID-19 total deaths
1073	Jefferson County	AL	259
1089	Madison County	AL	130
1097	Mobile County	AL	443
4013	Maricopa County	AZ	1364
4019	Pima County	AZ	226
4021	Pinal County	AZ	194
5119	Pulaski County	AR	213
6019	Fresno County	CA	244
6029	Kern County	CA	189
6037	Los Angeles County	CA	1633
6059	Orange County	CA	301
6065	Riverside County	CA	352
6067	Sacramento County	CA	435
6071	San Bernardino County	CA	312
6073	San Diego County	CA	311
6077	San Joaquin County	CA	215
8041	El Paso County	CO	170
10003	New Castle County	DE	138
13051	Chatham County	GA	146
13063	Clayton County	GA	141
13089	DeKalb County	GA	162
13121	Fulton County	GA	214
13135	Gwinnett County	GA	169
16001	Ada County	ID	155
16027	Canyon County	ID	124
17031	Cook County	IL	740
18097	Marion County	IN	270
20091	Johnson County	KS	130
20173	Sedgwick County	KS	165
21111	Jefferson County	KY	297
22019	Calcasieu Parish	LA	144
22033	East Baton Rouge Parish	LA	299
22051	Jefferson Parish	LA	228
22063	Livingston Parish	LA	152
22071	Orleans Parish	LA	176
22103	St. Tammany Parish	LA	163
26099	Macomb County	MI	176

26163	Wayne County	MI	382
27053	Hennepin County	MN	125
28047	Harrison County	MS	191
28049	Hinds County	MS	176
29077	Greene County	MO	182
29095	Jackson County	MO	381
29189	St. Louis County	MO	180
32003	Clark County	NV	1245
32031	Washoe County	NV	167
35001	Bernalillo County	NM	129
36047	Kings County	NY	379
36061	New York County	NY	132
36081	Queens County	NY	280
36103	Suffolk County	NY	145
37081	Guilford County	NC	122
37119	Mecklenburg County	NC	193
39035	Cuyahoga County	OH	172
39049	Franklin County	OH	145
39113	Montgomery County	OH	138
40109	Oklahoma	OK	209
40143	Tulsa County	OK	331
41019	Douglas County	OR	141
41029	Jackson County	OR	141
42003	Allegheny County	PA	184
42101	Philadelphia County	PA	246
45019	Charleston County	SC	138
45045	Greenville County	SC	253
45063	Lexington County	SC	180
45079	Richland County	SC	155
45083	Spartanburg County	SC	228
47065	Hamilton County	TN	127
47093	Knox County	TN	213
47157	Shelby County	TN	414
48027	Bell County	TX	231
48029	Bexar County	TX	1323
48061	Cameron County	TX	234
48113	Dallas County	TX	990
48121	Denton County	TX	184
48135	Ector County	TX	136
48167	Galveston County	TX	233
48201	Harris County	TX	2175
48215	Hidalgo County	TX	455
48245	Jefferson County	TX	218
48257	Kaufman County	TX	136

48303	Lubbock County	TX	167
48309	McLennan County	TX	204
48339	Montgomery County	TX	399
48355	Nueces County	TX	303
48439	Tarrant County	TX	834
48453	Travis County	TX	345
49035	Salt Lake County	UT	191
53011	Clark County	WA	150
53033	King County	WA	294
53063	Spokane County	WA	268

Table S2. Socio-economic characteristics of counties in the study. Note that personal income, population density, percent uninsured, percent of poverty and percent population over 65 years of each county were the statistical data in 2020. Data of cumulative vaccination rate was taken at August 1st, 2021 (median date in the study) and OxCGRT Government Response Index of counties was calculated as the average in study time.

	Mean (SD)	Maximum	75th percentile	Median	25th percentile	Minimum
Personal income (U.S.D)	57249.2 (18439.5)	191220.0	59939.0	55294.0	48434.0	31153.0
Vaccination rate (%)	43.3 (10.1)	67.2	49.6	43.3	38.9	13.8
Population density (per sq. mile)	2595.1 (8611.0)	71874.1	1891.6	776.9	354.8	22.0
Percent uninsured (%)	11.1 (4.7)	30.5	13.0	10.2	8.4	4.3
Percent of poverty (%)	15.5 (4.4)	531.2	17.4	15.2	12.7	5.6
Percent population over 65yrs (%)	14.9 (2.8)	26.3	16.3	14.9	13.1	9.6
OxCGRT government response index	40.9 (8.0)	53.1	45.6	42.8	33.9	23.4

Table S3. Spearman's rank order correlation coefficients between socio-economic characteristics of counties in study.

Spearman correlation coefficients	Personal income	Vaccination rate	Population density	Percent uninsured	Percent of poverty	Percent population over 65yrs	Oxford government response index
Personal income	1						
Vaccination rate	0.6835	1					
Population density	0.5789	0.4001	1				
Percent uninsured	-0.2269	-0.3108	-0.0523	1			
Percent of poverty	-0.3184	-0.1533	0.0098	0.3346	1		
Percent population over 65yrs	0.1762	0.3564	-0.0688	-0.3656	0.0446	1	
Oxford government response index	0.0661	-0.0242	-0.0340	-0.2436	0.2177	0.2896	1

Table S4. Significance tests for spearman's rank order correlation between socio-economic characteristics of counties in study.

P-value	Personal income	Vaccination rate	Population density	Percent uninsured	Percent of poverty	Percent population over 65yrs	Oxford government response index
Personal income	0						
Vaccination rate	< 0.001	0					
Population density	< 0.001	<0.001	0				
Percent uninsured	0.0247	0.0047	0.6088	0			
Percent of poverty	0.0014	0.1718	0.9233	< 0.001	0		
Percent population over 65yrs	0.0827	0.0011	0.5008	< 0.001	0.6629	0	
Oxford government response index	0.5178	0.8302	0.7393	0.0156	0.0313	0.0038	0

Table S5. The results of RR for heat (90th vs 50th percentiles of temperature distribution) with 95% CI in main model and sensitivity analysis.

Model setting	RR: tmean 90th vs 50th	If p-value < 0.05
Main model	1.108 (1.046, 1.173)	√
df of RH from 3 to 2	1.104 (1.042, 1.169)	√
df of time from 2 to 1	1.067 (1.012, 1.126)	√
df of time from 2 to 3	1.039 (0.989, 1.090)	
Internal knots set at the 50th and 75th percentiles	1.098 (1.040, 1.159)	√
Replacing with daily maximum temperature	1.082 (1.030, 1.136)	√
Taking 7-day weighted cumulation of COVID-19 deaths	1.091 (1.026, 1.160)	√
Maximum lag from 1 to 3	1.153 (1.071,1.242)	√
Changing RH to AH	1.196 (1.114, 1.284)	√
Replacing the exposure-response basis from B-spline to natural spline	1.103 (1.055, 1.153)	√
Replacing the exposure-response basis from B-spline to fourth-order polynomials	1.104 (1.057, 1.154)	√
**Add vaccination rates in partial counties with data:**		
Main model without vaccination rates	1.099 (1.042, 1.158)	√
Adjust for vaccination rates	1.057 (1.004, 1.113)	√
**Alternate ozone in partial counties with data:**		
Main model without ozone	1.103 (1.052,1.157)	√
Adjust for ozone	1.100 (1.048,1.154)	√

Supplementary Figures

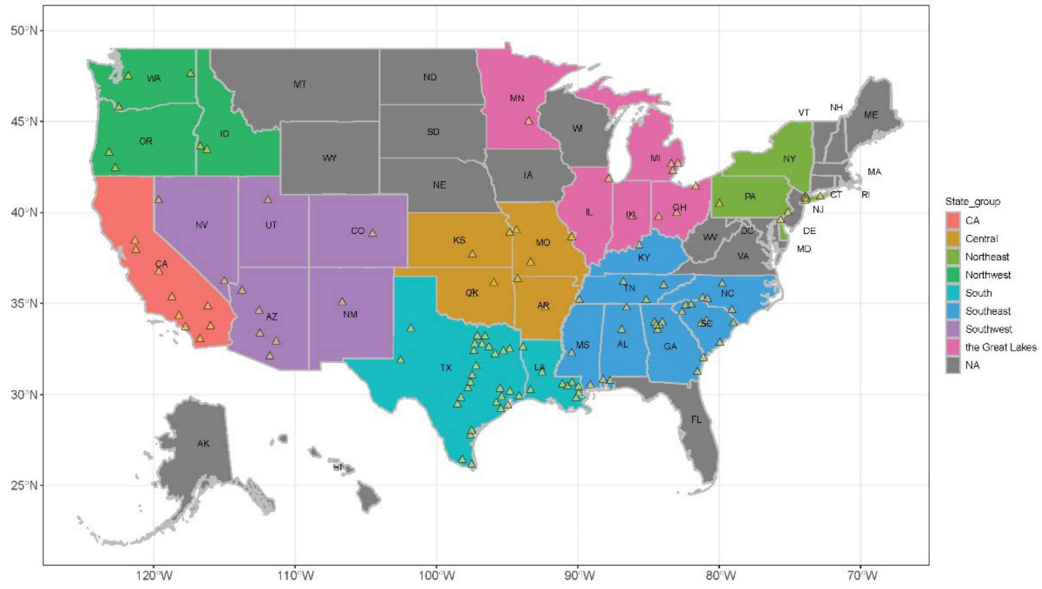

Figure S1. Geographical distribution of the counties with large number of COVID-19 deaths in the U.S. and spatial division in the multivariate meta-analysis. The yellow triangle represents the location of the counties with large counts of COVID-19 deaths in the study period, and the grey areas represent that no county is located in these states.

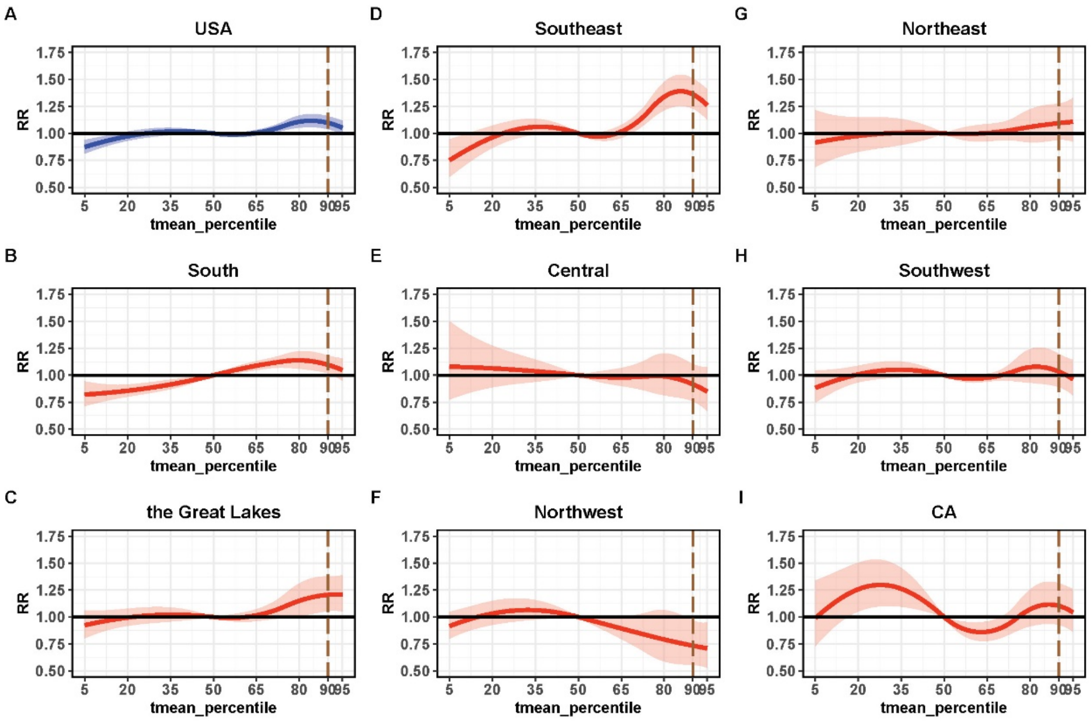

Figure S2. Overall cumulative temperature–mortality associations from multivariate meta-analysis by country and regions in sensitivity analysis (setting internal knots at the 50th and 75th percentiles of temperature compared with main model).

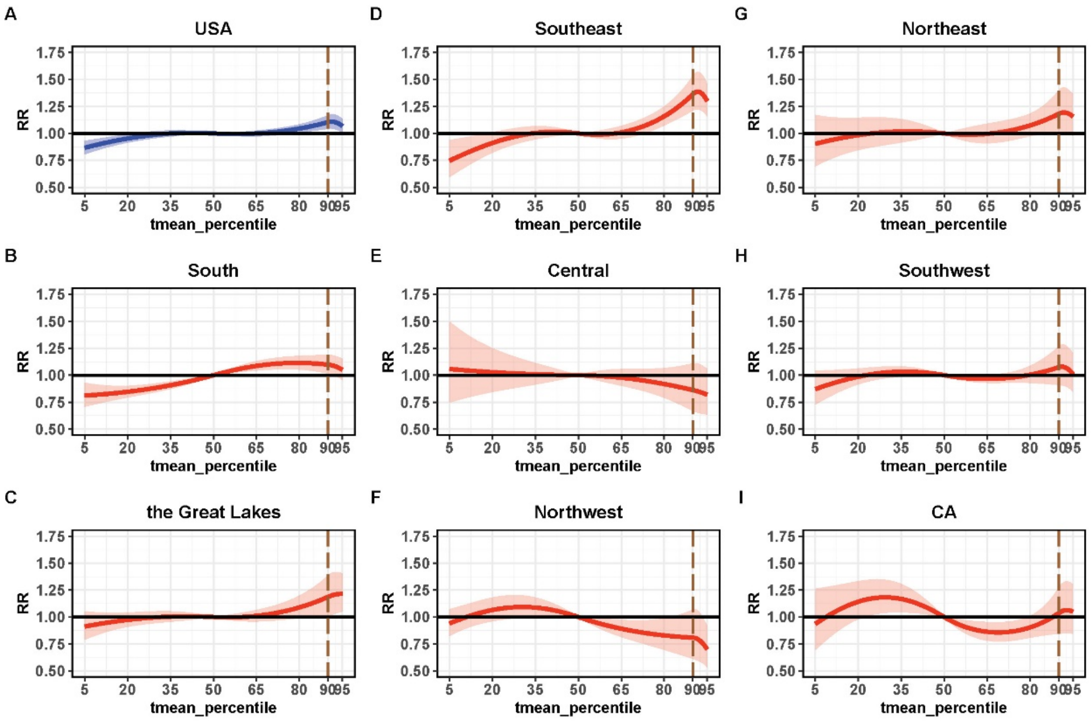

Figure S3. Overall cumulative temperature–mortality associations from multivariate meta-analysis by country and regions in sensitivity analysis (adjusting df of RH from 3 to 2 compared with main model).

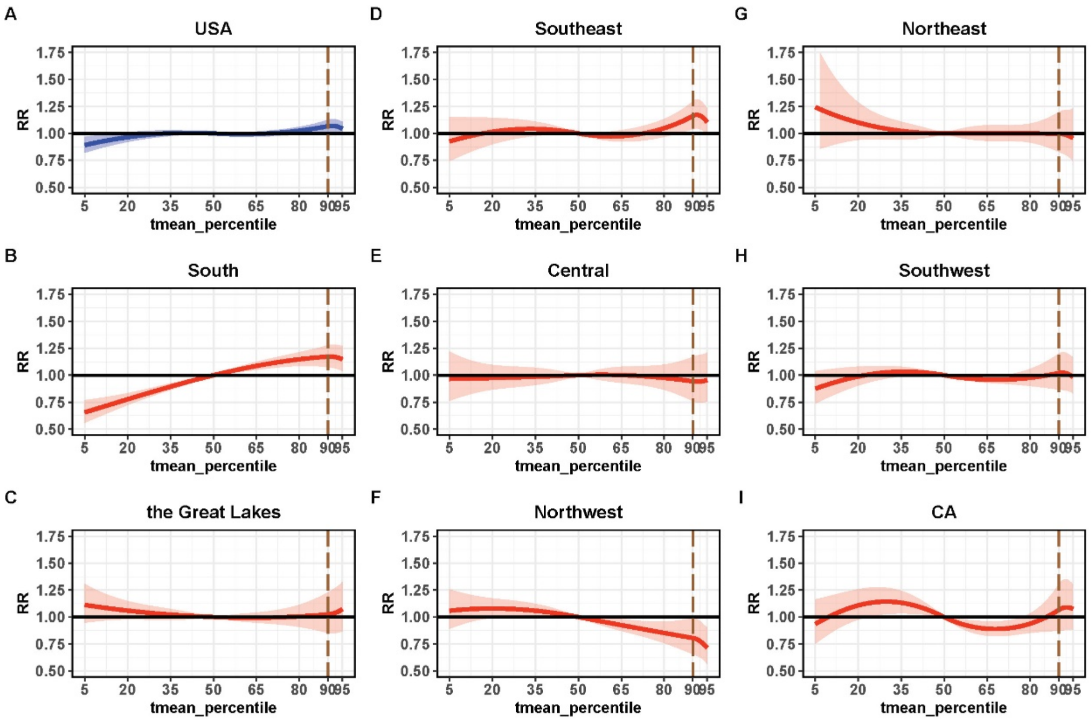

Figure S4. Overall cumulative temperature–mortality associations from multivariate meta-analysis by country and regions in sensitivity analysis (adjusting df of time from 2 to 1 compared with main model).

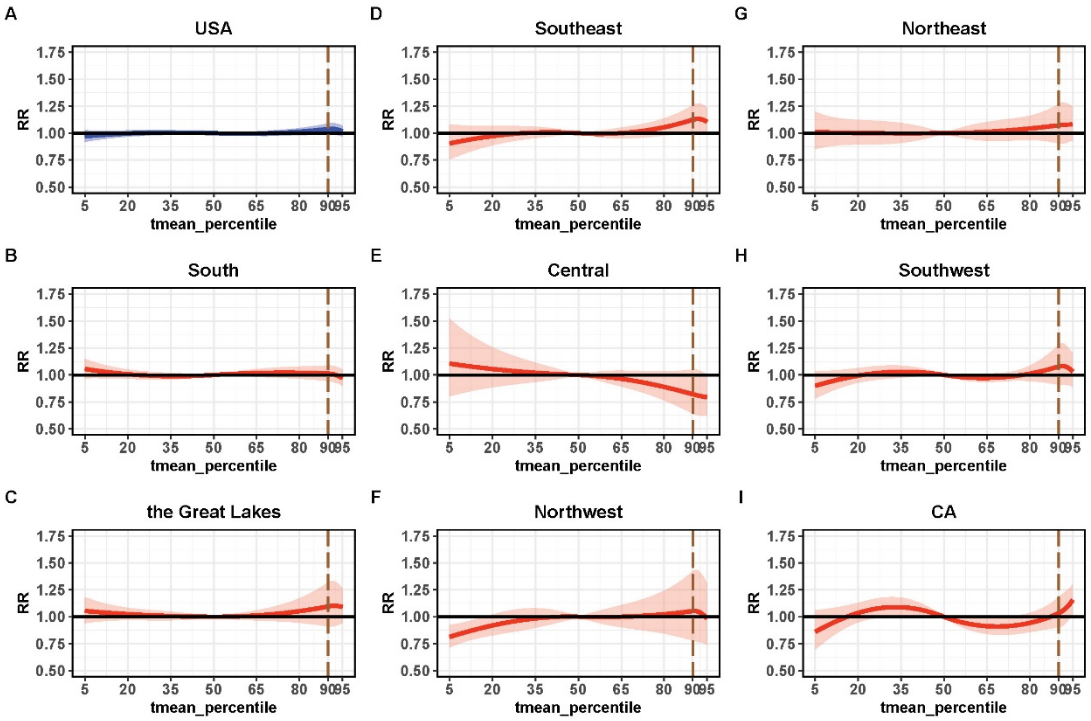

Figure S5. Overall cumulative temperature–mortality associations from multivariate meta-analysis by country and regions in sensitivity analysis (adjusting df of time from 2 to 3 compared with main model).

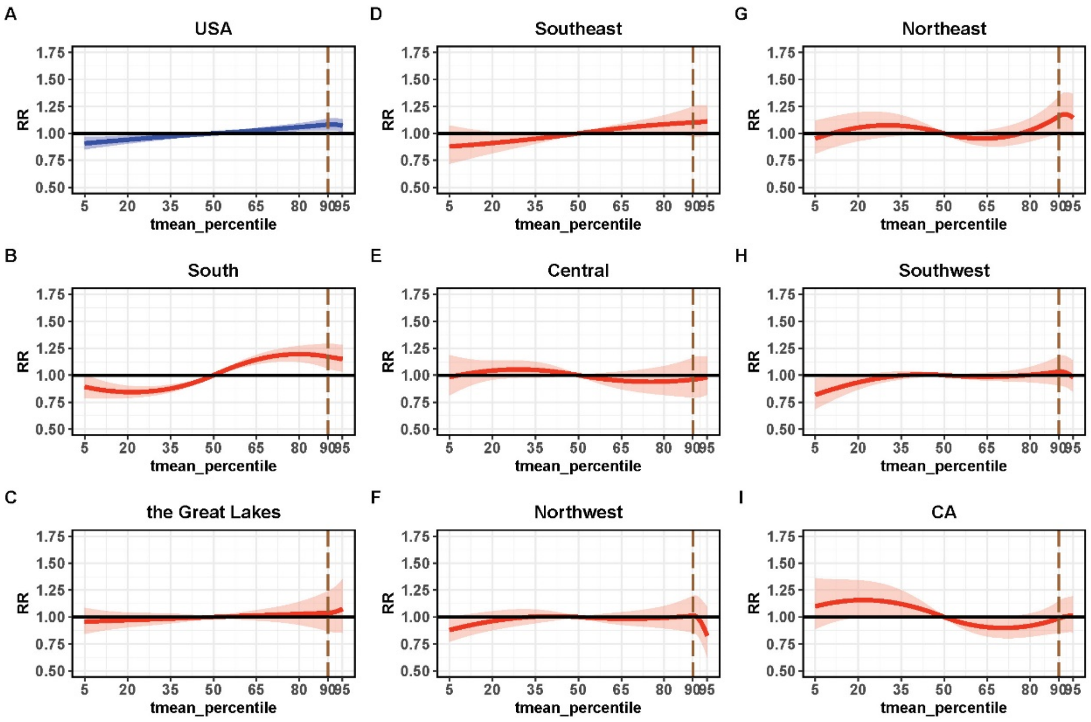

Figure S6. Overall cumulative temperature–mortality associations from multivariate meta-analysis by country and regions in sensitivity analysis (replacing daily mean temperature with daily maximum temperature compared with main model).

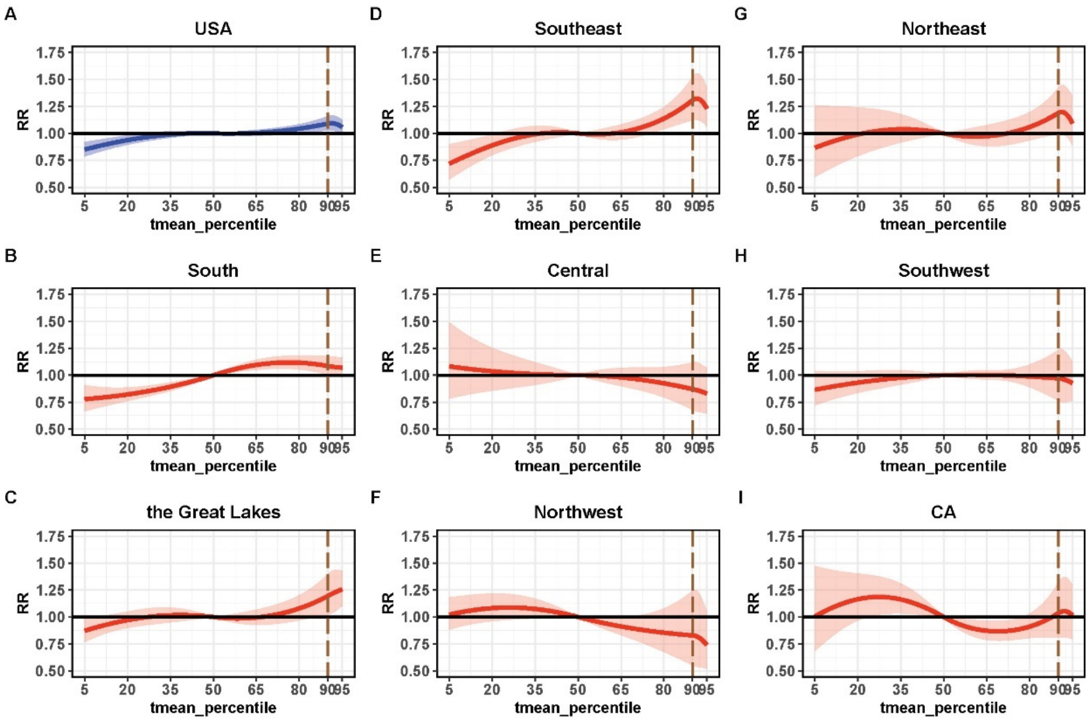

Figure S7. Overall cumulative temperature–mortality associations from multivariate meta-analysis by country and regions in sensitivity analysis (taking 7-day weighted cumulative deaths of COVID-19 as dependent variable compared with main model). The weighting factors were taken 1.4 in the deaths of first three days and 0.7 in the deaths of last four days to keep the sum of factors equal to 7.

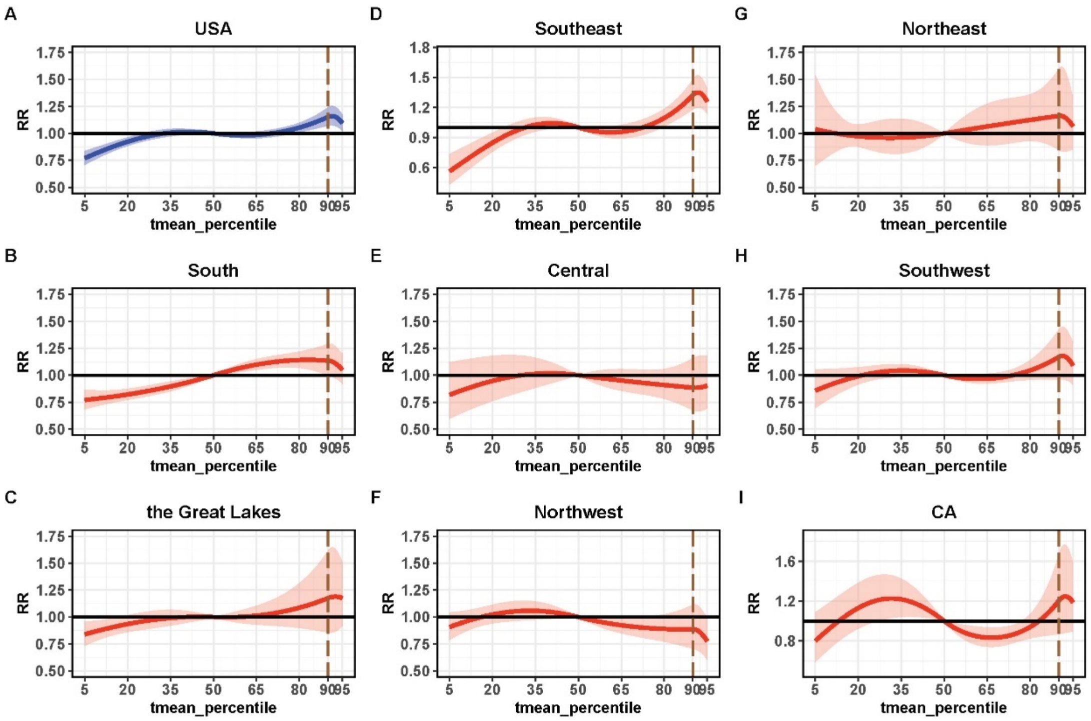

Figure S8. Overall cumulative temperature–mortality associations from multivariate meta-analysis by country and regions in sensitivity analysis (adjusting maximum temperature lag from 1 to 3 compared with main model).

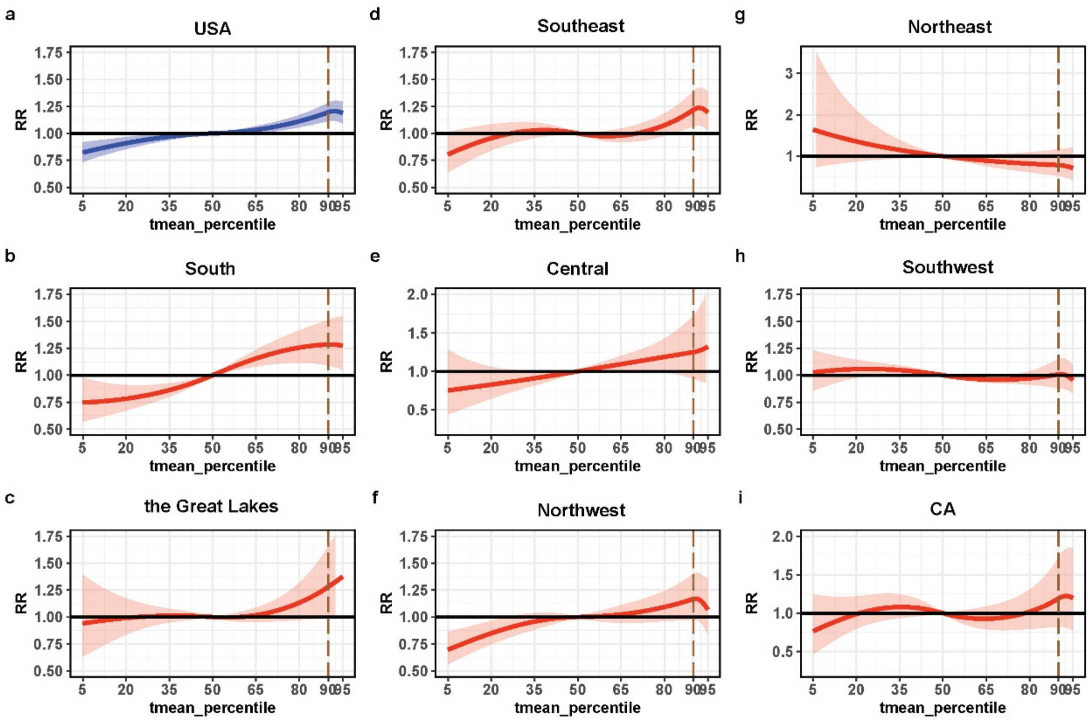

Figure S9. Overall cumulative temperature–mortality associations from multivariate meta-analysis by country and regions in sensitivity analysis (changing the RH to AH).

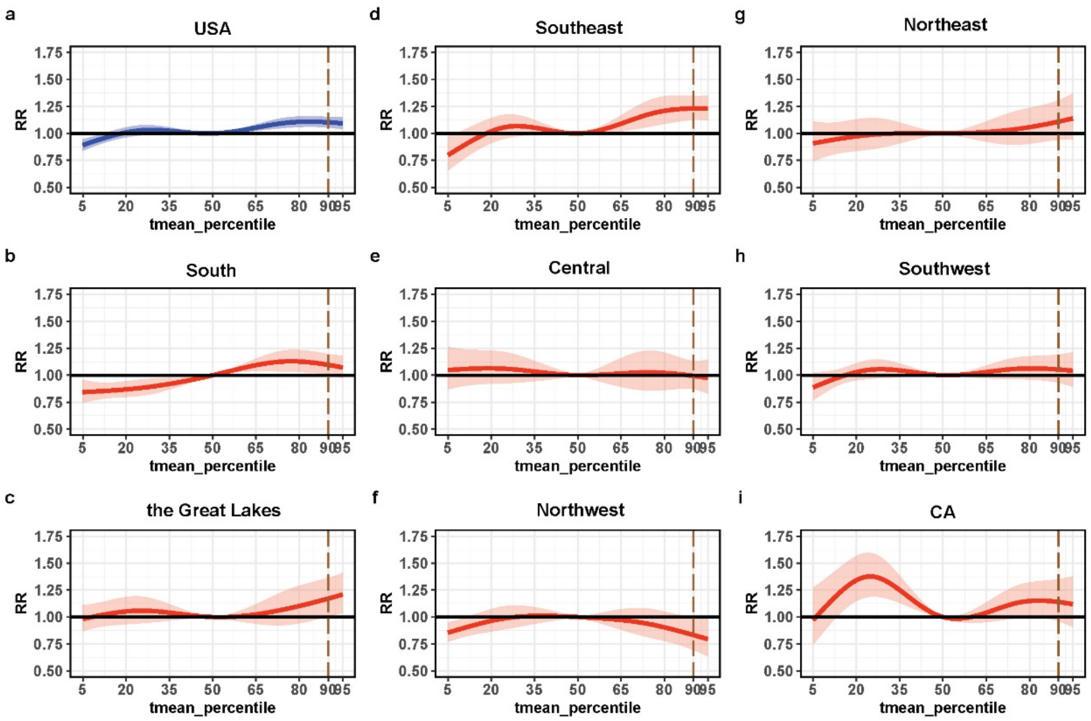

Figure S10. Overall cumulative temperature–mortality associations from multivariate meta-analysis by country and regions in sensitivity analysis (replacing the exposure-response basis from B-spline to natural spline).

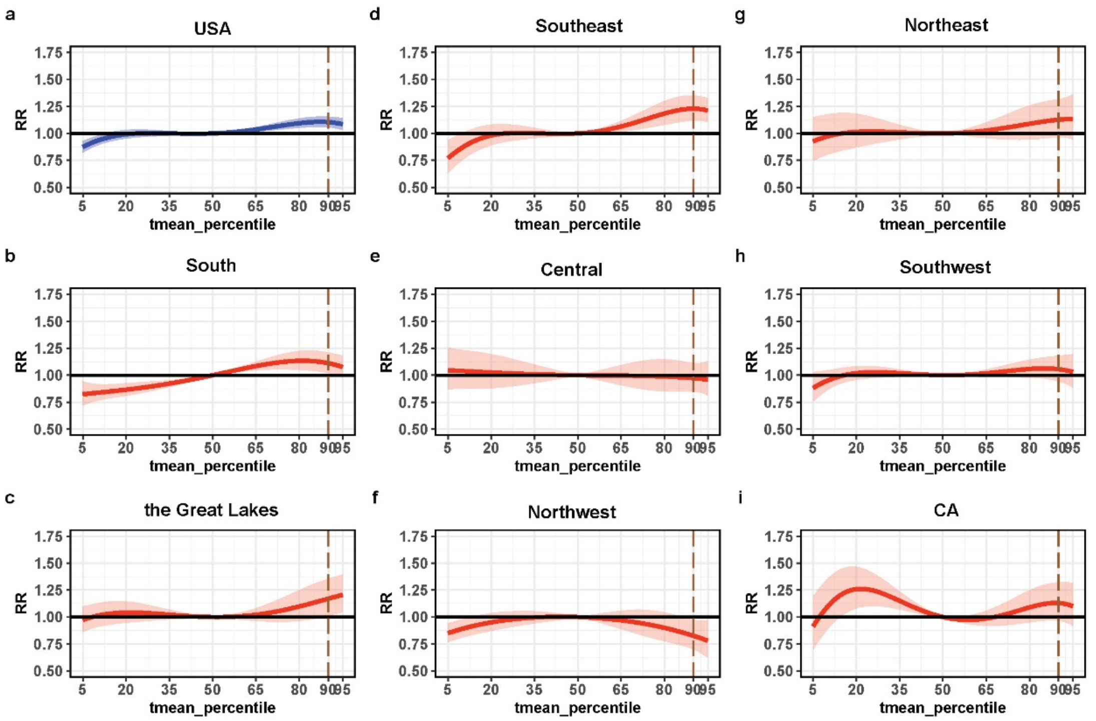

Figure S11. Overall cumulative temperature–mortality associations from multivariate meta-analysis by country and regions in sensitivity analysis (replacing the exposure-response basis from B-spline to fourth-order polynomials).

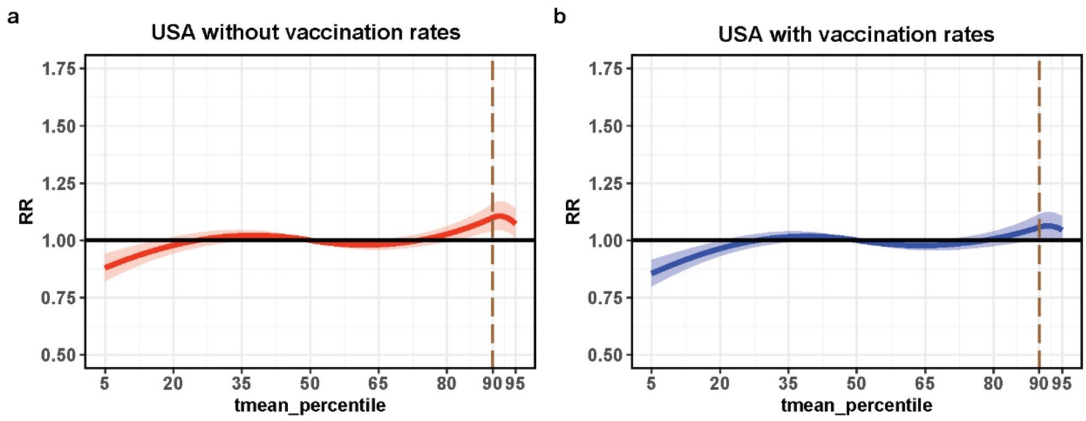

Figure S12. Overall cumulative temperature–mortality associations from multivariate meta-analysis by county A) without daily changes of vaccination rates and B) with daily changes of vaccination rates in sensitivity analysis. Note that the sensitivity analyses were conducted only on partial counties compared to the main model since updating data of vaccination rates was not available in Texas.

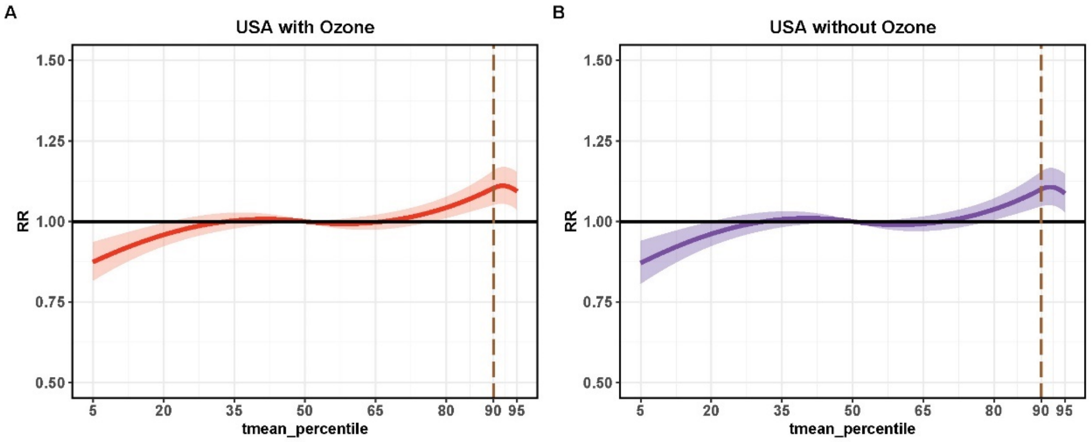

Figure S13. Overall cumulative temperature–mortality associations from multivariate meta-analysis by county A) with daily ozone and B) without daily ozone in sensitivity analysis. Note that the sensitivity analyses were conducted only on partial counties compared to the main model since ozone data was not available in all of the research areas (81 counties with data).
